# Efficacy of Daptomycin-Containing Regimen for Treatment of Staphylococcal or Enterococcal Vertebral Osteomyelitis: A Prospective Clinical Experience

**DOI:** 10.3390/antibiotics9120889

**Published:** 2020-12-10

**Authors:** Alessandro Russo, Giancarlo Ceccarelli, Valeria Bellelli, Luigi Bianchi, Federica Marincola Cattaneo, Fabrizio Gregori, Valeria Palmarini, Nicola Marotta, Alessandro Landi, Alessandro Cuzzolino, Matteo Stefanini, Alessandro Aureli, Claudio Maria Mastroianni, Mario Venditti, Gabriella d’Ettorre, Francesco Sabetta

**Affiliations:** 1 Department of Clinical and Experimental Medicine, University of Pisa, Via Paradisa 2, 56124 Pisa, Italy; 2 Internal Medicine Unit, Policlinico Casilino, 00169 Rome, Italy; vbellelli.polcas@eurosanita.it (V.B.); lbianchi.polcas@eurosanita.it (L.B.); fmcattaneo.polcas@eurosanita.it (F.M.C.); fsabetta.polcas@eurosanita.it (F.S.); 3 Department of Public Health and Infectious Diseases, “Sapienza” University of Rome, 00185 Roma, Italy; giancarlo.ceccarelli@uniroma1.it (G.C.); claudio.mastroianni@uniroma1.it (C.M.M.); mvenditti@policlinicoumberto1.it (M.V.); gabriella.dettorre@uniroma1.it (G.d.); 4 Spinal Surgery Unit, San Carlo di Nancy Hospital—GVM Care and Research, 00165 Roma, Italy; fgregori@gvmnet.it (F.G.); vpalmarini@gvmnet.it (V.P.); nmarotta@gvmnet.it (N.M.); dott.alessandro.landi@gmail.com (A.L.); 5 Interventional Radiology, Policlinico Casilino, 00169 Rome, Italy; acuzzolino.polcas@eurosanita.it (A.C.); mstefanini.polcas@eurosanita.it (M.S.); 6 Orthopedic Unit, Policlinico Casilino, 00169 Rome, Italy; aaureli.polcas@eurosanita.it

**Keywords:** vertebral osteomyelitis, daptomycin, source control of infection, biofilm, clinical failure

## Abstract

Vertebral osteomyelitis (VO) is a compelling clinical entity for clinicians, because of its insidious and indolent course that makes diagnosis difficult. A concern is reported about the choice of antibiotic regimens, duration of therapy, and criteria to switch to oral therapy. We conducted a prospective observational study. All consecutive hospitalized patients with a confirmed diagnosis of VO caused by staphylococcal or enterococcal strains were analyzed. The primary endpoint was the analysis of clinical cure at the end of therapy. A propensity score for receiving therapy with daptomycin was added to the model. During the study period, 60 episodes of confirmed VO were observed. The main etiology of infection was methicillin-resistant Staphylococcus aureus (29%). Overall, clinical failure at end of therapy was reported in 11 (18.3%) patients. Logistic regression analysis, after propensity score, showed that >2 vertebrae involved (OR 2.4, CI95% 1.12–5.24, *p* = 0.002) and inadequate drainage of infection (OR 4.8, CI95% 2.45–8.51, *p* < 0.001) were independently associated with failure of therapy, while the use of a daptomycin-containing-regimen (OR 0.15, CI 95% 0.04–0.46, *p* < 0.001) with clinical cure. VO caused by staphylococcal or enterococcal strains is associated with an important rate of clinical failure. Daptomycin-containing regimen was strongly associated with clinical cure. Considering that over 70% of VO etiology is caused by Gram-positive strains but the etiology of infection is obtained in about 75% of cases, these data may help physicians to choose the appropriate antibiotic regimen.

## 1. Introduction

Vertebral osteomyelitis (VO) represents for clinicians a fascinating infection to manage and treat, because of its insidious and indolent course. The diagnosis is frequently difficult and can often be delayed for several months and initially be misdiagnosed and mismanaged because signs and symptoms were not interpreted as spondylodiscitis. The etiology of infection is globally obtained in about 50% of cases, making the choice of antimicrobial regimen and duration of therapy challenging; however, a standardized approach may be associated with a higher rate of defined etiology >70% [1,2,3]. 

The use of an image-guided biopsy is recommended in patients with high suspicion of VO and if the microbiologic diagnosis has not been established by serologic tests and/or blood cultures (BC) [4]. Of interest, in the last years, the role of positron emission tomography (PET) imaging has been assessed, considering also its high sensitivity for detecting osteomyelitis. ^18^F-FDG PET/CT can help to confirm or exclude the presence of metabolically active infection in these patients and guide their appropriate treatment [5,6,7].

Empiric antimicrobial therapy should be withheld, when possible, until a microbiologic diagnosis is confirmed; however, over 70% of VO etiology is caused by Gram-positive strains, mainly staphylococci. Treatment of VO can be challenging as antibiotics should penetrate through the rigid bone structure. Several pharmacokinetic studies measured the extent of penetration of different antibiotics into the bone and joint tissues [8]. Then, a concern is reported about the choice of antibiotic regimen, the duration of therapy, and, not less importantly, the criteria to switch to oral therapy. Of importance, results from a recent study showed non-inferiority of oral antibiotics versus intravenous antibiotics for the treatment of bone and joint infections [9].

The aim of the study was the analysis of clinical features, antibiotic regimen, and outcome of patients with confirmed VO caused by staphylococci or enterococci. 

## 2. Results

### 2.1. Baseline Characteristic and Etiology of VO

**Figure 1:**
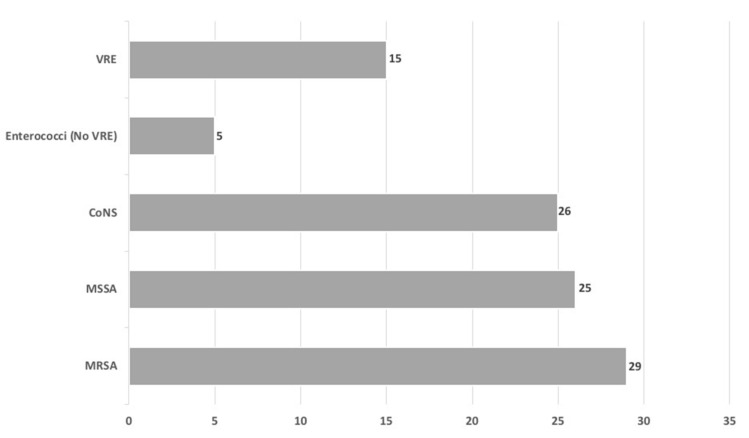
Etiology of VO (%). **Legend.** VO: vertebral osteomyelitis; MRSA: methicillin-resistant *Staphylococcus aureus;* MSSA: methicillin-sensitive *Staphylococcus aureus;* CoNS: coagulase-negative staphylococci; VRE: vancomycin-resistant enterococci.

During the study period, 60 episodes of confirmed VO were observed. As reported in Figure 1, etiology of infection (obtained from BC and/or vertebral biopsy/drainage) was: methicillin-resistant *Staphylococcus aureus* (MRSA, 29%), followed by methicillin-sensitive *Staphylococcus aureus* (MSSA, 26%), coagulase-negative staphylococci (CoNS, 25%), vancomycin-resistant enterococci (VRE, 15%). 

Overall, clinical failure at end of therapy was reported in 11 (18.3%) patients: in 5 (45.4%) patients was observed relapse of infection, in 4 (36.4%) persistence of infection, while in 2 (18.2%) patients was reported 30-day mortality. 

Table 1 reports baseline characteristics and outcomes of the study population along with univariate analysis of risk factors for clinical cure or failure. Mean age was 65.2 years, 14-day antimicrobial treatment discontinuation before vertebral biopsy was obtained in 35 (58.3%) patients, concomitant endocarditis was observed in 5 (18.3%) of cases; biopsy/drainage of infection was performed in 42 (70%) patients with an inadequate source control of infection reported in 8.3% of cases. Univariate analysis of risk factors for clinical cure or failure showed that >2 vertebrae involved (100% vs. 63.2%, *p* = 0.024), inadequate source control of infection (45.4% vs. 0%, *p* < 0.001), and a longer time to biopsy (5.9 vs. 3.8 days, *p* = 0.019) were more frequently observed in patients with failure of therapy, compared with clinical cure group.

### 2.2. Comparison between Patients with Clinical Cure or Failure during Study Period

Univariate analysis comparing antibiotic regimens in definitive therapy among patients with clinical cure or failure of therapy is reported in Table 2. Daptomycin-containing regimen was used exclusively in patients with clinical cure (46.9% vs. 0%, *p* = 0.004). A higher length of definitive antibiotic therapy (44.9 vs. 38.3 days, *p* < 0.001), and time to oral therapy (41.9 vs. 21.9 days, *p* < 0.001) were more frequently observed in patients with a clinical failure of therapy. 

### 2.3. Comparison between Antibiotic Regimens Used in Definitive Therapy

Table 3 reports a univariate analysis comparing patients treated with daptomycin-containing regimen or other antibiotic regimens in definitive therapy. An antimicrobial therapy in the previous 30 days (86.9% vs. 56.7%, *p* = 0.021), >2 vertebrae involved (95.6% vs. 54%, *p* < 0.001) were more frequently reported in patients treated with daptomycin in definitive therapy. 

**Figure 2:**
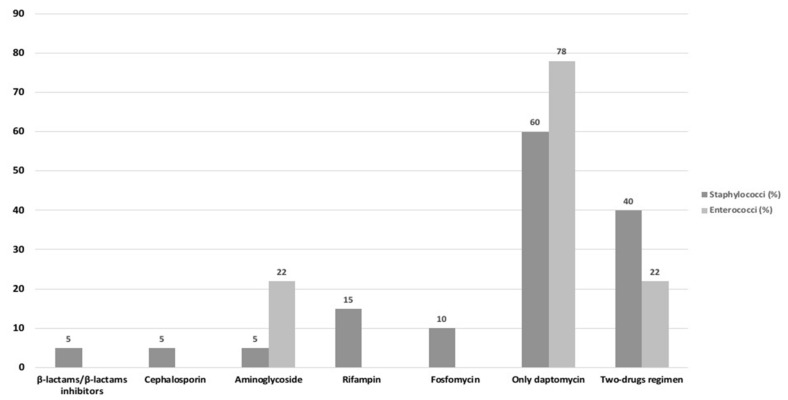
Antibiotics in combination with daptomycin for definitive therapy (%).

The antibiotics used in combination with daptomycin for definitive therapy are reported in Figure 2. Overall, daptomycin was used in monotherapy for 60% and 78% of VO caused, respectively, by staphylococcal and enterococcal strains. For enterococcal VO, daptomycin was exclusively used with aminoglycosides; for staphylococcal etiology with rifampin (15%), followed by fosfomycin (10%). In all cases, daptomycin was given at 8–10/mg/Kg dosage.

Patients with clinical failure were treated with the following antibiotic regimens in definitive therapy: linezolid for VRE (1 patient), levofloxacin followed by vancomycin for MSSA (4 patient), vancomycin for MRSA (2 patients), amoxicillin/clavulanate plus rifampin for MSSA (2 patients), vancomycin plus rifampin for MRSA (1 patient), teicoplanin for MSSA (1 patient).

### 2.4. Variables Independently Associated with Clinical Failure or Cure

Logistic regression analysis showed that >2 vertebrae involved (OR 2.4, CI95% 1.12–5.24, *p* = 0.002) and inadequate drainage of infection (OR 4.8, CI95% 2.45–8.51, *p* < 0.001) were independently associated with failure of therapy, while the use of a daptomycin-containing-regimen (OR 0.15, CI 95% 0.04–0.46, *p* < 0.001) with clinical cure (see Table 4). After adjustment for the propensity score in the logistic regression model evaluating risk factors for mortality, all the variables remained in the model without significant differences.

## 3. Discussion

Our data showed that VO caused by staphylococcal or enterococcal strains is associated with an important rate of clinical failure. Of importance, while >2 vertebrae involved, and an inadequate drainage of infection were associated with treatment failure, the use of daptomycin-containing regimens in definitive therapy was associated with clinical cure as confirmed by propensity score for receiving therapy with daptomycin. 

VO is very difficult to diagnose and treat infection, considering also the high rate of patients on antibiotic therapy at the time of diagnosis. This observation results in a low rate of positivity from BC and vertebral biopsy to determine the etiology of infection, causing a high rate of empiric therapy [10,11]. In our study population, failure of therapy for VO was observed in 11 (18.3%) patients, in line with the failure rates of treated patients with VO in most clinical studies that vary between 10% and 30% [4,12]. Of interest, no specific etiology of infection resulted associated with clinical failure at logistic regression analysis.

Therapeutic management of spondylodiscitis in patients with treatment failure should be customized to the suspected reasons for therapeutic failure. Consultation with an infectious disease physician and a surgeon experienced in the management of VO should be warranted in these patients with suspected or confirmed treatment failure. The decision about surgical intervention should be individualized [13]. As a matter of fact, inadequate drainage of abscess is reported as an important risk factor for treatment failure in these patients [7,14]. 

Monitoring systemic inflammatory markers, such as C-reactive protein (CRP), and follow-up with magnetic resonance imaging (MRI) are mandatory to assess the adequate response to therapy, clarify the presence of abscess to drain, and identify spinal instability that could benefit from surgery. In previous studies, patients with evidence at MRI of epidural and/or paraspinal soft tissue infection appear to be at a greater risk for treatment failure, if not adequately and on time treated [15]. However, it is important to underline that the frequency and utility of obtaining a follow-up with inflammatory markers (like CRP) during antimicrobial therapy for VO have not been definitely established. MRI has an important role to assess the response of soft tissue infection, but bone and disk abnormalities typically appear to worsen in the first weeks, even in successfully treated patients [16,17]. FDG-PET/TC could be used in association with MRI for the detection of VO but can also contribute to the early determination of the response to therapy [18,19].

To our knowledge, this is the first study reporting the favorable use of daptomycin for VO in a large series of patients. Previous experiences about the use of daptomycin for VO reported a higher rate of clinical cure, compared to other antibiotic regimens [20,21]. In a retrospective analysis, the use of daptomycin resulted in a significantly higher rate of cure compared with that of vancomycin [22,23]. Vancomycin use in the setting of difficult-to-treat infections, such as endocarditis and osteomyelitis, was associated with a high failure rate [24]. The reasons for a higher failure rate with vancomycin included limited bone penetration, limited activity in microbial biofilm environment, and lack of bactericidal activity. As a matter of fact, biofilm formation is an integral part of the pathophysiology of osteomyelitis [25] and considering that daptomycin has shown good activity in biofilm environment, either as monotherapy or in antibiotic combination, its use seems to be reasonable [26,27,28]. Daptomycin concentrations highly exceed the MIC_90_ of Gram-positive pathogens causing osteomyelitis and septic arthritis [29,30,31,32]. In a previous study, a mean daptomycin bone penetration rate of about 15% was observed in the bone of infected patients, after 8 mg/kg dose [33]. Additionally, in our series, all patients were treated with a dosage of 8–10 mg/Kg.

Some important limitations of this study should be highlighted. Firstly, the observational design of the study; second, the short follow-up period (6 months) reduces generalizability and comparability of these results. Finally, no therapeutic drug monitoring was available in all patients.

## 4. Materials and Methods 

### 4.1. Study Design

We conducted a prospective observational study among a large teaching-hospital and two 300-bed hospitals in Rome, Italy. From January 2018 to June 2020, all consecutive hospitalized patients with a confirmed diagnosis of VO caused by staphylococcal or enterococcal strains and with a minimum follow-up period of 6 months were analyzed. The study was preapproved by the local ethics committee and conducted in accordance with the principles of the Declaration of Helsinki.

All patients were managed and treated according to a predefined protocol [7].

### 4.2. Data Collection and Definitions

Data of patients were prospectively extracted, using a standard form, from medical records, computerized hospital databases, and/or clinical charts. Demographics, clinical radiological and laboratory findings, comorbidities, and Charlson Comorbidity Index, microbiological findings, previous surgery (if performed), source of infection, septic shock, duration of hospital stay and definitive antibiotic therapy, reasons for clinical treatment failure, and 30-day mortality were collected.

The following standard definitions were established prior to data analysis: *relapse*—a new diagnosis of VO caused by the same organism after clinical and microbiological resolution of a previous episode treated VO; *time to biopsy*—the number of days that elapsed between diagnosis of VO and vertebral biopsy (when performed); *persistent infection*—patients still treated for VO at three month follow-up without resolution of the described symptoms; *clinical treatment failure*—lack of response to the definitive antimicrobial regimen, as reflected by the presence of any of the following after ≥45 days of therapy: ongoing fever, leukocytosis, or other clinical signs of infection that could not be attributed to causes other than VO; *clinical cure*—response to the definitive antimicrobial regimen, as reflected after ≥45 days of therapy by the absence of any symptom (i.e., lumbar pain), fever, leukocytosis, and reduction >75% of CRP plus improvement of radiological findings at MRI and/or PET-CT; *30-day mortality*—death from any cause within the 30 days following diagnosis of VO [7]. 

The presence of other foci of infection was identified by means of clinical and imaging examinations. Fluid collections and abscesses were drained and the fluid cultured when were identified at CT scan, MRI, or PET-CT. Adequate control of the source of infection was defined as the drainage of fluid collections and abscesses associated with VO within 48 h after the diagnosis of VO: inadequate drainage of infection was classified if the drainage of vertebral abscesses or other fluid collections has not been performed within 48 h.

Patients who were diagnosed with VO based on imaging study and/or blood or tissue culture results were treated according to the IDSA (Infectious Diseases Society of America) guideline for VO [4]. 

All patients were evaluated at baseline and every month for at least 12 months; patients in which was documented a clinical cure in the first 6 months were not reevaluated. 

### 4.3. Microbiological Identification

Etiology of infection was obtained accordingly with local laboratory techniques. The bacterial pellet from positive cultures was used for MALDI-TOF MS (Bruker Daltonics, Billerica, MA, USA) identification and for molecular analysis. The SensiTitre™ system (Thermo Fisher Scientific, Waltham, MA, USA) or the Vitek 2 automated system (bioMérieux, Marcy l’Etoile, France) were used for antimicrobial susceptibility testing. Minimum inhibitory concentrations (MICs) were established according to the European Committee on Antimicrobial Susceptibility Testing (EUCAST) breakpoints [34]. 

### 4.4. Antimicrobial Treatment Evaluation

Empiric and definitive antibiotic regimens were selected according to clinical judgment by infectious disease specialists and were subsequently modified according to microbiological results.

Depending on the number of drugs used (1 or >1), treatment regimens were classified either as monotherapy or combination therapy. Empiric antibiotic therapy (antimicrobial chemotherapy implemented within 24 h after the onset of infection) was assessed along with definitive antibiotic therapy (antimicrobial treatment based on in vitro isolate susceptibility). Antibiotics analyzed in definitive therapy were administered for at least 50% of the total duration of therapy. The time to initial definitive therapy was the period between the diagnosis of infection and initial definitive therapy.

### 4.5. Primary Endpoints and Statistical Analysis 

Primary endpoint of the study was the analysis of clinical cure at the end of therapy.

The differences between groups were evaluated using the chi-squares test or Fisher’s exact test (for categorical variables) and the two-tailed *t*-test or Mann–Whitney test (for continuous variables), when appropriate. Univariate and multivariate analyses were used to determine the effects of different variables on clinical treatment failure or clinical cure in this study population. A propensity score for receiving therapy with daptomycin was added to the model. The propensity score was calculated using a nonparsimonious multivariate logistic regression model in which the outcome variable was the treatment with daptomycin. Possible confounding factors and interactions were weighted during analysis with a backward stepwise selection and considering *p* ≤ 0.05 for all variables, to determine the effects of all variables on outcomes. All reported *p*-values are two-tailed, wald confidence intervals and odd ratios were computed based on estimated standard errors. All analyses were done with the SPSS software package (version 20.0, SPSS Inc., Chicago, IL, USA). 

## 5. Conclusions

In conclusion, VO caused by staphylococcal or enterococcal strains is associated with an important rate of clinical failure. The number of vertebrae involved and inadequate drainage of infection are important determinants of therapeutic failure. Our data showed that a daptomycin-containing regimen was strongly associated with clinical cure. Considering that over 70% of VO etiology is caused by Gram-positive strains but etiology of infection is obtained in about 75% of cases, these data may help physicians to choose the appropriate antibiotic regimen, especially in patients without a confirmed etiology of infection, in order to early evaluate also the criteria to switch to oral therapy [7,35]. However, randomized clinical trials are mandatory to confirm or to exclude these observations.

## Figures and Tables

**Table 1:** Baseline characteristics and outcome of study population and univariate analysis of risk factors for clinical cure or failure.

Variables	All Population *n* = 60 (%)	Clinical Cure *n* = 49 (%)	Clinical Failure *n* = 11 (%)	*p*
Age, mean ± SD (years)	65.2 ± 19.9	66.4 ± 20.7	64.9 ± 19.4	0.654
Male sex	39 (65)	31 (63.2)	8 (72.7)	0.713
Charlson Comorbidity Index, mean ± SD	2.2 ± 1.9	2.2 ± 1.8	2 ± 1.9	1.0
Previous spinal surgery	35 (58.3)	31 (63.2)	4 (36.3)	0.1
Previous antimicrobial therapy (30 days)	41 (68.3)	30 (61.2)	11 (100)	**0.011**
14-day antimicrobial treatment discontinuation before vertebral biopsy	35 (58.3)	25 (51)	10 (90.9)	**0.018**
>2 vertebrae involved	42 (70)	31 (63.2)	11 (100)	**0.024**
Endocarditis	5 (8.3)	4 (8.1)	1 (9.1)	1.0
Other localization of infection	11 (18.3)	7 (14.2)	4 (36.3)	0.1
Biopsy/drainage performed	42 (70)	37 (75.5)	5 (45.4)	0.07
Blood culture positivity	35 (58.3)	28 (57.1)	7 (63.6)	0.748
CRP concentration (mg/L), mean ± SD	76.2 ± 41.5	77.3 ± 40.2	74.9 ± 44.9	0.098
WBC concentration (10^9^/L), mean ± SD	9.7 ± 5.9	9.9 ± 6.2	9.2 ± 5.2	0.787
Inadequate source control of infection	5 (8.3)	0	5 (45.4)	**<0.001**
Time to biopsy, mean ± SD (days)	4.6 ± 2.8	3.8 ± 3.3	5.9 ± 2.6	**0.019**
Length of hospitalization, mean ± SD (days)	36.5 ± 13.8	35.8 ± 14.5	39.7 ± 11.8	**0.032**
Use of FDG-PET/CT	4 (6.6)	3 (6.1)	1 (9.1)	0.565

**Legend.** CRP: C-reactive protein; WBC: white blood cells; FDG-PET/CT: F18-fluorodeoxyglucose positron emission tomography/computed tomography.

**Table 2:** Univariate analysis comparing antibiotic regimens in definitive therapy among patients with clinical cure or failure of therapy.

Antibiotic Therapy *	Clinical Cure *n* = 49 (%)	Clinical Failure *n* = 11 (%)	*p*
Use of only 1 antibiotic	25 (51)	7 (63.6)	0.518
Use of 2 antibiotics in combination	22 (44.9)	4 (36.3)	0.741
Use of 3 antibiotics in combination	2 (4.1)	0	1.0
Daptomycin-containing regimen	23 (46.9)	0	**0.004**
Vancomycin-containing regimen	12 (24.4)	7 (63.6)	0.07
Teicoplanin-containing regimen	4 (8.1)	1 (9.1)	1.0
β-lactams-containing regimen	5 (10.2)	2 (18.2)	0.601
Fosfomycin-containing regimen	4 (8.1)	0	1.0
Quinolones-containing regimen	5 (10.2)	4 (36.3)	**0.049**
Dalbavancin-containing regimen	3 (6.1)	0	1.0
Linezolid-containing regimen	2 (4.1)	1 (9.1)	0.461
Rifampin-containing regimen	6 (12.2)	3 (27.2)	0.344
Length of definitive antibiotic therapy, mean ± SD (days)	38.3 ±12.9	44.9 ± 23.1	**<0.001**
Time to initial definitive therapy, mean ± SD (days)	8.9 ± 5.2	10.9 ± 4.9	0.091
Time to oral therapy, mean ± SD (days)	21.9 ± 8.3	41.9 ± 19.3	**<0.001**

* dosages according to “The Sanford Guide to Antimicrobial Therapy 2020”. **Legend.** SD: standard deviation.

**Table 3:** Univariate analysis comparing patients treated with daptomycin-containing regimen or other antibiotic regimens in definitive therapy.

Variables	Other Antibiotic Regimens *n* = 37 (%)	Daptomycin-Containing Regimen *n* = 23 (%)	*p*
Age, mean ± SD (years)	65 ± 17.5	63.9 ± 18.4	0.324
Male sex	22 (59.4)	17 (73.9)	0.281
Charlson Comorbidity Index, mean ± SD	2.3 ± 1.8	2.2 ± 1.9	1.0
Previous spinal surgery	19 (51.3)	16 (69.5)	0.188
Previous antimicrobial therapy (30 days)	21 (56.7)	20 (86.9)	**0.021**
14-day antimicrobial treatment discontinuation before vertebral biopsy	20 (54)	15 (65.2)	0.431
>2 vertebrae involved	20 (54)	22 (95.6)	**<0.001**
Endocarditis	3 (8.1)	2 (8.7)	1.0
Other localization of infection	4 (10.8)	7 (30.4)	0.086
Biopsy/drainage performed	23 (62.1)	19 (82.6)	0.147
Blood culture positivity	19 (51.3)	16 (69.5)	0.188
CRP concentration (mg/L), mean ± SD	77.4 ± 39.5	76. 8 ± 37.8	1.0
WBC concentration (10^9^/L), mean ± SD	9.3 ± 5.8	9.8 ± 4.9	0.876
Inadequate source control of infection	1 (2.7)	4 (17.4)	0.066
Time to biopsy, mean ± SD (days)	5.1 ± 3.1	4.2 ± 3.8	0.234
Length of hospitalization, mean ± SD (days)	42.4 ± 23.8	28.4 ± 13.9	**<0.001**
Use of FDG-PET/CT	3 (8.1)	1 (4.3)	1.0
Clinical failure	11 (29.7)	0	**0.004**
30-day mortality	2 (5.4)	0	0.519

**Legend.** CRP: c-reactive protein; WBC: white blood cells; FDG-PET/CT: F18-fluorodeoxyglucose positron emission tomography/computed tomography.

**Table 4:** Multivariate analysis of risk factors for clinical failure among patients with VO.

Variables	OR	CI 95%	*p*	OR	CI 95%	*p*
	Without Propensity Score Adjustment			With Propensity Score Adjustment		
Daptomycin-containing-regimen	0.15	0.04–0.46	<0.001	0.3	0.15–0.87	0.001
>2 vertebrae involved	2.4	1.12–5.24	0.002	3.22	2.09–5.47	<0.001
Inadequate drainage of infection	4.8	2.45–8.51	<0.001	5.4	3.22–9.12	<0.001

**Legend.** VO: vertebral osteomyelitis.
